# Identification of TRPV4 as a novel target in invasiveness of colorectal cancer

**DOI:** 10.1186/s12885-021-08970-7

**Published:** 2021-11-23

**Authors:** Peng Zhang, Jian Xu, Hua Zhang, Xiao-Yu Liu

**Affiliations:** 1 Longgang E.N.T. Hospital & Shenzhen Key Laboratory of E.N.T., Institute of E.N.T, No. 3004 Longgang Avenue, Shenzhen, Guangdong China; 2 School of Medicine, Jiangnan University, Wuxi, Jiangsu China; 3 School of Medicine, Southern University of Science and Technology, 1088 Xueyuan Avenue, Shenzhen, Guangdong China

**Keywords:** Colon cancer, TRPV4, Invasiveness, ZEB1

## Abstract

**Background:**

Emerging evidence has indicated the critical role of TRPV4 in diverse human cancers. However, the underlying molecular mechanism of TRPV4 in colon cancer invasiveness is still unknown.

**Methods:**

Immunohistochemistry staining was used to analyze the expression of TRPV4 and ZEB1 in clinical tissues; Wound healing and transwell assays were applied to determine the cell invasiveness; Western blot was used to explore the relation between TRPV4 and ZEB1.

**Results:**

Colon cancer cells were transfected with siRNA against TRPV4 or HC067047 (a selective TRPV4 antagonist), TRPV4 full-length plasmid or siRNA against ZEB1, or both, in order to measure cell migration and invasion. And we found that TRPV4 silencing or inhibition exhibited an inhibitory role in colon cancer cell migration and invasion, coupled with compromised EMT process, and suppressed AKT activity. TRPV4 stimulated expression of ZEB1 and consequently contributed to EMT process and invasiveness. It was also revealed that overexpression of TRPV4 and ZEB1 in clinical patients with local metastasis, and positive correlation between TRPV4 and ZEB1.

**Conclusions:**

Our results uncovered the role of TRPV4 in tumor metastasis and highlighted the potential mechanism of TRPV4-ZEB1 axis in indicating EMT.

**Supplementary Information:**

The online version contains supplementary material available at 10.1186/s12885-021-08970-7.

## Background

Colorectal cancer (CRC) is the third most common human malignancy and the fourth crucial cause of cancer-related death in worldwide [1–6]. CRC metastasis is the main cause of mortality in patients [7–9]. However, the underlying pathogenic mechanisms in CRC metastasis are still not entirely clear. Therefore, there is an urgent need to develop novel therapeutic strategies based on the molecular mechanisms of metastatic spread of CRC.

Transient receptor potential vanilloid type 4 (TRPV4) is a Ca^2+^-permeable nonselective cation channel that senses heat, mechanical forces and arachidonic acid [10]. TRPV4 is not only a key player in cardiovascular system but also in cancer [11, 12]. Aberrant expression of TRPV4 is involved in diverse human cancers including colon cancer [13]. Our recent study has demonstrated that TRPV4 is associated with colon cancer development [13]. Our results illustrated that TRPV4 promotes colon cancer progression via inhibition of PTEN signaling [13]. However, TRPV4 has not been previously shown to be involved in colon cancer invasiveness, and the underlying mechanism has not been reported.

The epithelial-mesenchymal transition (EMT) can promote cancer cell losing cell polarity and cell-cell adhesion, resulting cell detachment, migration, and invasion [14]. During EMT process, the expression of E-cadherin, as an epithelial marker, is down-regulated, while the mesenchymal markers including Vimentin and N-cadherin are increased [15]. It is well known that ZEB transcription factor families play important role in regulating EMT [16].

In this study we explored the role of TRPV4 in CRC invasiveness in vitro and in vivo. Our findings indicated that TRPV4 exerts these functions through activating the AKT signaling pathway, regulating the expression of ZEB1 in CRC cells, and then modulating EMT. Thus, our study indicates that TRPV4 can functionally regulate EMT, suggesting a novel link between TRPV4 and colon cancer aggressiveness.

## Methods

### Ethics statement and patients

The Review Board of the Affiliated Hospital of Jiangnan University approved the clinical samples for research purposes (NO.LS2018020). This study was confirmed to the principles contained in the World Medical Association Declaration of Helsinki. Informed consent was requested as anonymous specimens and was given by all human participants in this study. The human colorectal cancer samples (*n* = 106, without chemical treatments, paraffin embedding tissues) were randomly collected from Affiliated Hospital of Jiangnan University. Patients were recruited between 2010 and 2012.

### Cell culture

The human colon cancer cell lines SW620 was purchased from the American Type Culture Collection (ATCC, VA, USA). HCT-116 and HT-29 were purchased from the Chinese Academy of Sciences cell bank (CBCCCAS, Shanghai, China). Cell lines were maintained in McCoy’s 5A or Leibovitz’s L-15 medium (Thermo Fisher Scientific, MA, USA) supplemented with 10% fetal bovine serum (Thermo Fisher Scientific, MA, USA), 100 U/mL penicillin, and 100 μg/mL streptomycin (Thermo Fisher Scientific, MA, USA). Cells were cultured at 37 °C with 5% CO_2_ in a humidified incubator.

### RT-qPCR analysis

Total RNA was isolated by Trizol (Invitrogen, CA, USA). Reverse transcription polymerase chain reaction (RT-PCR) was performed with the PrimeScript™ RT reagent kit (Takara, China) following the manufacturer’s instructions. qPCR was performed using QuantiNova SYBR Green PCR Master Mix (QIAGEN, Hilden, Germany) in the LightCycler 480 Real Time PCR system (Roche, Basel, Sweden). Primer sequences are listed in the supplemental Table S1.

### siRNA transfection

Cells in 12-well plate were transiently transfected with 25 nM siRNA using DharmaFECT 1 Transfection Reagent (GE Dharmacon, CO, USA) according to the manufacturer’s instructions. Briefly, adding 5 μL of 5 μM siRNA to 95 μL of serum free medium in tube 1. Adding 5 μL of DharmaFECT 1 Transfection Reagent to 95 μL of serum free medium in tube 2. Gently mix and incubate for 5 min. Then add the contents of tube1 to tube 2, and add 800 μL of antibiotic-free complete medium for a total volume of 1000 μL transfection medium. TRPV4 silencing was performed using siRNAs targeting the following sequences: siRNA#1: 5′-AUCUUGGUAACAAACUUGG-3′, and siRNA#2: ON-TARGET plus SMART pool against human TRPV4 siRNA (GE Dharmacon, CO, USA). ZEB1 silencing was performed using siRNA targeting the following sequences: 5′-CAGUGUUCCAUGCUUAAGA-3′. AKT silencing was performed using siRNA targeting the following sequences: 5′-GCACCUUC AUUGGCUACAATT-3′.

### Plasmid transfection

The plasmid carrying human TRPV4 (GenBank NM_021625.4) was obtained from GeneCopoeia, Inc. Cells were transfected with the TRPV4 plasmid (TRPV4) or Vector using Lipofectamine™ 2000 reagent (Invitrogen, Carlsbad, CO, USA) according to the manufacturer’s instructions.

### Western blot analysis

Western blots were performed as previously described [17]. Primary antibodies against ACTB (Santa, TX, USA), TRPV4 (alomone labs, Israel), ZEB1, E-cadherin, N-cadherin and Vimentin (Cell Signaling Technology, MA, USA) were incubated at 4 °C overnight with constant shaking. HRP labeled secondary antibodies (Cell Signaling Technology, MA, USA) were incubated at room temperature for 1 h.

### Wound healing assay

The wound healing assay was used a monolayer denudation assay as described previously [18]. Briefly, cells cultured in 12-well plates as confluent monolayers were mechanically scratched using a 200 μL pipette tip to create the wound. Cells were washed with PBS to remove the detached cells and then cultured to allow migration. Photographs were taken and the percent of wound closure was calculated.

### Migration and invasion assays

Cell migration assay was determined using the transwell chamber (Corning) as described previously [18]. Briefly, cells were harvested, washed, resuspended and then seeded into the upper chamber in serum-free medium. The medium containing 10% FBS was placed in the lower chamber and the cells were further incubated for 24 h, cells in the upper chamber were removed with a cotton swab, and the rest of the membrane had invaded the cells. Cells migrated through the membrane were fixed with 4% paraformaldehyde and stained with crystal violet. Cell invasion was determined with Matrigel matrix (BD Biosciences) coated on the upper surface of the transwell chamber. Cells were seeded, fixed and stained as described in migration assay.

### Immunohistochemistry staining

Immunohistochemistry staining was performed as previously described [19]. Primary antibodies against TRPV4 (ACC-034, alomone labs, Israel) and ZEB1(#70512, Cell Signaling Technology, MA, USA) were used. All staining was assessed by pathologists blinded to the origin of the samples and patient outcomes. The widely-accepted German semi-quantitative scoring system was used to assess the staining intensity and proportion of stained cells. Each specimen was assigned a score according to the intensity of staining (0, none; 1, weak; 2, moderate; 3, strong) and the proportion of stained cells (0, 0%; 1, 1–24%; 2, 25–49%; 3, 50–74%; 4, 75–100%). The final score for immunoreactivity was determined by multiplying the intensity by the proportion, ranging from 0 to 12.

### Statistical analyses

Statistical analysis was performed using two-tailed Student’s t-test or one-way ANOVA. All analyses were carried out with the GraphPad Prism software version 5.0 (GraphPad Software, CA, USA). Data were expressed as mean ± standard error of the mean (SEM) of at least three independent experiments. The difference was considered significant if *p* value< 0.05.

## Results

### Inhibition of TRPV4 expression or activity suppresses colon cancer cell migration and invasion

**Fig. 1:**
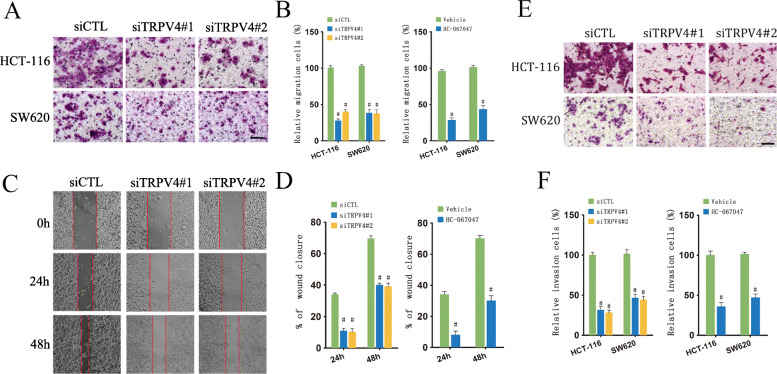
Inhibition of TRPV4 suppresses colon cancer cell migration and invasion. **A** and **B** representative images and summary data of the migration assay in HCT-116 cells and SW620 cells transfected with siCTL, siTRPV4#1 and siTRPV4#2 or treated with Vehicle or HC-067047, scale bar: 50 μm. **C** and **D** representative images and summary data of the wound healing assay in HCT-116 cells and SW620 cells transfected with siCTL, siTRPV4#1 and siTRPV4#2 or treated with Vehicle or HC-067047. **E** and **F** Representative images and summary data from invasion assay in HCT-116 cells and SW620 cells transfected with siCTL, siTRPV4#1 and siTRPV4#2 or treated with Vehicle or HC-067047, scale bar: 50 μm. Values represent the mean ± SEM, #, *p* < 0.05 compared to siCTL or Vehicle

To determine the effect of inhibition of TRPV4 on invasiveness in CRC cells, we performed migration assays using transwell in HCT-116 and SW620 cells. TRPV4 siRNAs were used to knockdown the expression of TRPV4 [13]. Pharmacological inhibiting TRPV4 was performed using HC-067047, a selective TRPV4 antagonist [13]. Our data showed that HCT-116 and SW620 cells exhibited suppressed migration when transfected with TRPV4 siRNAs when compared to cells that were transfected with control siRNA (Fig. 1A and B). We further observed that inhibition of TRPV4 activity by HC067047 decreased cell migration of HCT-116 and SW620 cells (Fig. 1B). In line with these results, HCT-116 cells exhibited a reduced migration capability at 24 h and 48 h when treated with TRPV4 siRNAs (Fig. 1C and D). Furthermore, we assessed HCT-116 and SW620 cell invasion using transwells that were coated with Matrigel. The results of the invasion assay indicated that HCT-116 and SW620 cells treated with TRPV4 siRNAs or HC-067047 had a decreased invasion ability when compared with control cells (Fig. 1E and F). Moreover, TRPV4 silencing or inhibition also suppressed migration and invasion in HT-29 cells (Supplementary Fig. S1). Taken together, these findings strongly indicated that TRPV4 inhibition suppressed cell invasiveness in CRC cells.

### TRPV4 overexpression promotes colon cancer cell migration and invasion

**Fig. 2:**
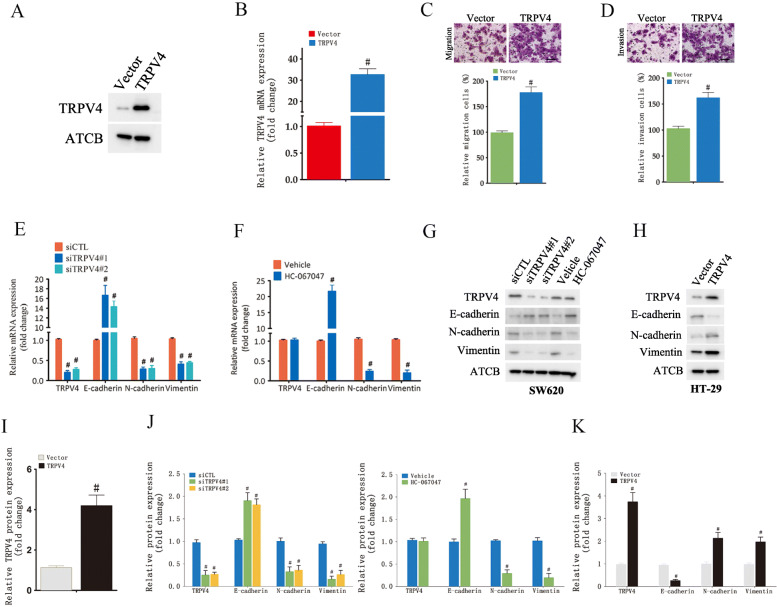
TRPV4 promotes colon cancer cell migration and invasion through regulating epithelial-mesenchymal transition process. **A** Western blot analysis of TRPV4 showing increased TRPV4 levels in HT-29 cells transfected with a TRPV4-overexpression construct. **B** TRPV4 mRNA levels in HT-29 cells transfected with a TRPV4-overexpression construct. **C** and **D** representative images and summary data of migration and invasion assay in HT-29 cells transfected with a TRPV4-overexpression construct, scale bar: 50 μm. **E** and **F** RNA levels of the epithelial-mesenchymal transition (EMT) markers in SW620 cells transfected with siCTL, siTRPV4#1 and siTRPV4#2 or treated with Vehicle or HC-067047. **G** Western blot analysis for EMT markers showing a decreased EMT process in SW620 cells transfected with siCTL, siTRPV4#1, and siTRPV4#2 or treated with Vehicle or HC-067047. **H** Western blot analysis for EMT markers showing an increased EMT process in HT-29 cells transfected with a TRPV4-overexpression construct. **I** Summary data for A. **J** Summary data for G. **K** Summary data for H. Values represent the mean ± SEM, #, *p* < 0.05 compared to siCTL or Vehicle or Vector

To further explore the role of TRPV4 in cell migration and invasion in CRC cells, HT-29 cells were transfected with full-length human TRPV4. Our data showed that both TRPV4 mRNA and protein levels were increased in TRPV4-overexpression cells (Fig. 2A, B and I). HT-29 cells exhibited an increased migration capability when transfected with full-length human TRPV4 when compared to cells which were transfected the vector plasmid (Fig. 2C). Consistent with these results, in the invasion assay, HT-29 cells that overexpressed TRPV4 showed markedly enhanced invasion capacity when compared to control cells (Fig. 2D). Moreover, TRPV4 overexpression also increased migration and invasion in HCT-116 and SW620 cells (Supplementary Fig. S2). Taken together, these findings suggested that TRPV4 might promote cell invasiveness in CRC cells.

### TRPV4 inhibition attenuates the EMT process in CRC cells

In previous studies, it was shown that EMT played an important role in normal development and cancer metastasis, induced a reversible change in cell phenotype, and resulted in mesenchymal features [20]. Consequently, we explored whether TRPV4 was involved in regulating EMT. After pharmacologic inhibition with HC-067047 or gene-silencing with TRPV4 siRNA, SW620 and HT-29 cells showed increased mRNA expression of the epithelial marker E-cadherin and reduced expression of mesenchymal markers N-cadherin and Vimentin (Fig. 2E and F and Supplementary Fig. S3). Western blot analysis further confirmed that E-cadherin was upregulated at the protein level and that protein levels of N-cadherin and Vimentin were downregulated (Fig. 2G and J). Not surprisingly, our data showed that in TRPV4-overexpressing HT-29, HCT-116 and SW620 cells, levels of E-cadherin were decreased and levels of N-cadherin and Vimentin were increased (Fig. 2H and K and Supplementary Fig. S4). Together, these findings strongly suggested that TRPV4 indicated EMT to promote CRC metastasis.

### TRPV4 increased ZEB1 expression to indicate EMT

**Fig. 3:**
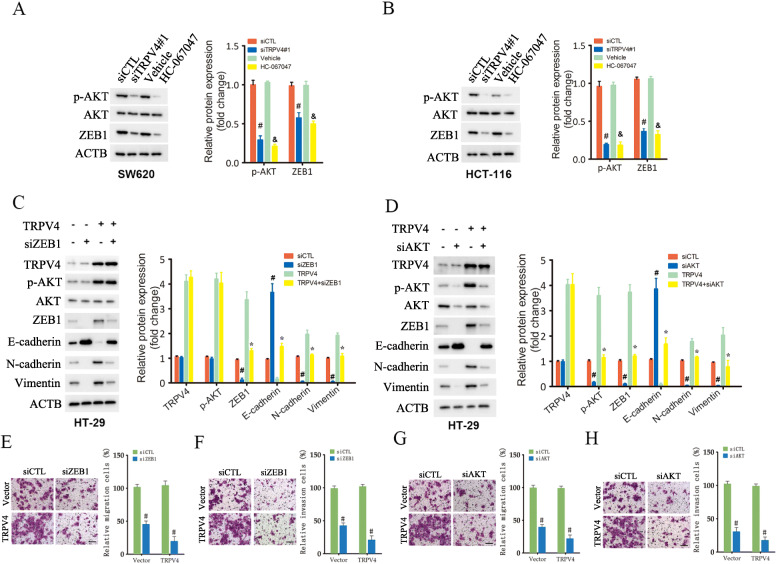
TRPV4 increases ZEB1 expression to enhance epithelial-mesenchymal transition. **A** and **B** Western blot analysis for p-AKT, AKT, ZEB1 and ATCB in HCT-116 and SW620 cells transfected with siCTL and siTRPV4#1 or treated with Vehicle or HC-067047. **C** and **D** representative images and summary data of Western blot demonstrating the effects of ZEB1 siRNA or AKT siRNA on the EMT process induced by TRPV4 overexpression in HT-29 cells. **E** and **F** representative images and summary data from migration and invasion assay demonstrating the effects of ZEB1 siRNA on TRPV4 overexpression induced cell motility, scale bar: 50 μm. **G** and **H** representative images and summary data from migration and invasion assay demonstrating the effects of AKT siRNA on TRPV4 overexpression induced cell motility, scale bar: 50 μm. Values represent the mean ± SEM, #, *p* < 0.05 compared to siCTL, &, *p* < 0.05 compared to Vehicle, *, *p* < 0.05 compared to TRPV4

In previous studies, it was demonstrated that transcriptional factors, including Twist, ZEB, and Snail families induced EMT, and that ZEB1 played a critical role in the regulation of EMT in CRC [21, 22]. Therefore, in this study, we evaluated whether TRPV4 regulated the expression of ZEB1 in CRC cells. TRPV4 knockdown significantly suppressed the expression of ZEB1 in HCT-116 and SW620 cells (Fig. 3A). Increasing evidence has suggested that AKT signaling could activate ZEB1 transcription [18]. Indeed, we confirmed that p-AKT expression was reduced after TRPV4 silencing or inhibition (Fig. 3A). To further confirm that TRPV4 regulated EMT through ZEB1, we transfected ZEB1 siRNA in TRPV4-overexprssing HT-29 cells. As shown in Fig. 3B, knockdown of ZEB1 increased the expression of E-cadherin and decreased levels of N-cadherin and Vimentin expression in TRPV4-overexprssion HT-29 cells. In addition, migration assay and invasion assay showed that ZEB1 silencing reversed TRPV4-overexprssion induced HT-29 cell invasiveness (Fig. 3C and D). To investigate the possible role of AKT in controlling TRPV4-regulated ZEB1 expression, TRPV4-overexprssing HT-29 cells were treated with AKT siRNA. Notably, knockdown of AKT reduced p-AKT levels, suppressed the expression of ZEB1, N-cadherin, and Vimentin, and increased the expression of E-cadherin in TRPV4-overexprssing HT-29 cells (Fig. 3E). Moreover, treatment with siRNA that targeted AKT also attenuated the increase in migration and invasion in TRPV4-overexprssing HT-29 cells (Fig. 3F and G). Taken together, these data suggested that TRPV4 indicated the EMT process through upregulating ZEB1 expression, which may be dependent on AKT signaling in CRC cells.

### TRPV4 expression is correlated with tumor metastasis in human colon cancer specimens

**Fig. 4:**
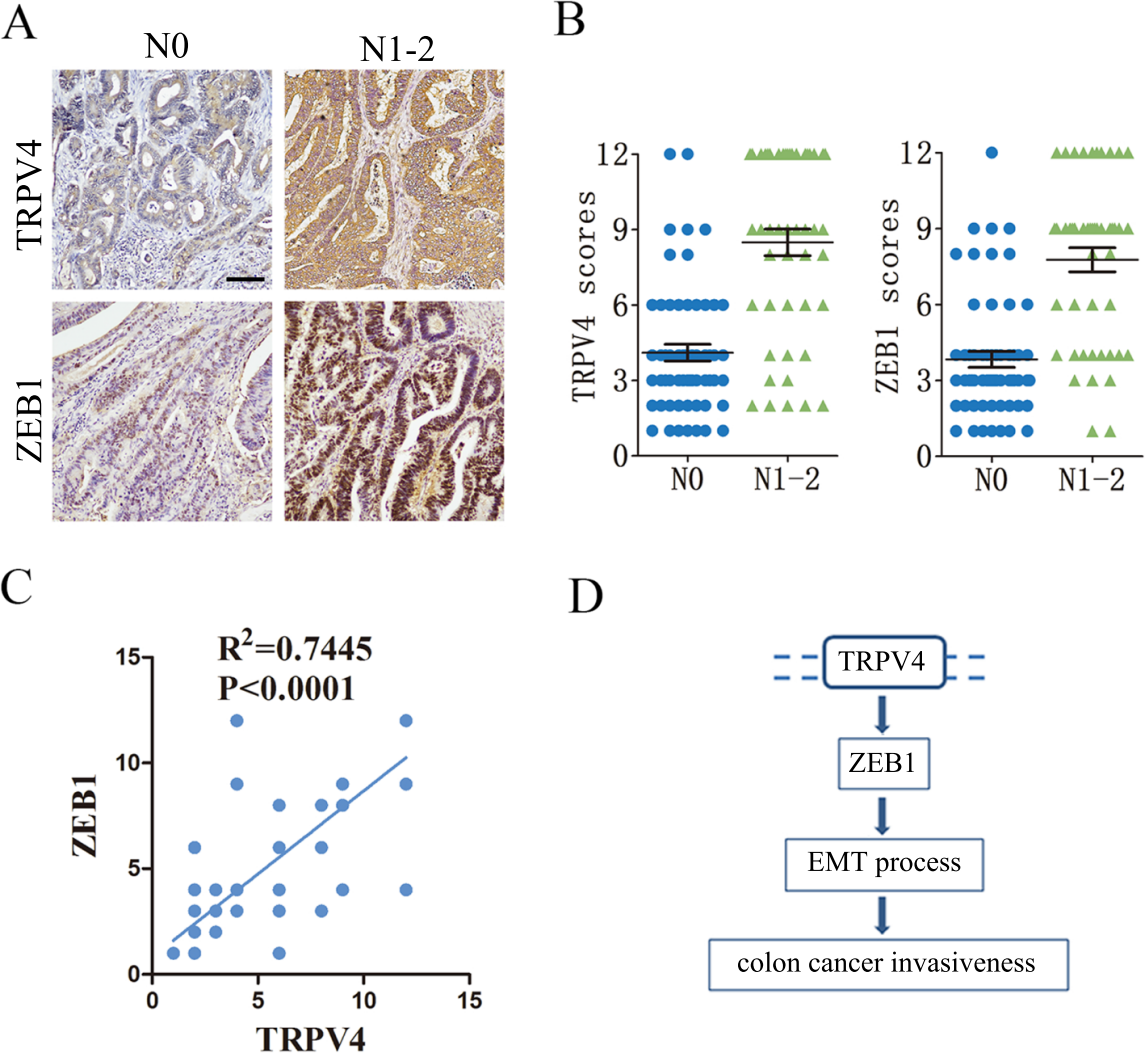
TRPV4 expression is correlated with tumor metastasis in human colon cancer specimens. **A** and **B** representative images and summary data of immunohistochemical staining of TRPV4 and ZEB1 in patients with or without lymph node metastasis (N0 *n* = 59, N1–2 *n* = 47) Scale bar: 50 μm. **C** Pearson correction of TRPV4 expression with ZEB1 (*n* = 106), data were analyzed using the Pearson correlation test. **D** Signaling connections involved in the TRPV4-modulated epithelial-mesenchymal transition (EMT) process in human colon cancer

To determine the clinical relevance between TRPV4 and tumor metastasis in CRC, we investigated 106 CRC specimens with or without local metastasis to lymph-nodes (Table 1). Immunohistochemical (IHC) staining showed that TRPV4 protein expression was mainly observed in the cytoplasm, and ZEB1 protein expression was mainly observed in the nucleus (Fig. 4A). As shown in Fig. 4A and B, the expression of TRPV4 and ZEB1 was elevated in CRC specimens with local metastasis (N1–2) compared to CRC specimens without local metastasis (N0). Furthermore, the expression of TRPV4 was significantly and positively correlated with ZEB1 expression, which suggested the mechanistic relevance and potential regulatory relationship between TRPV4 and ZEB1 in clinical specimens (Fig. 4C). Together, these results indicated that TRPV4 may play a key role in CRC metastasis.

**Table 1:** Demographic information of CRC specimens used for the IHC analysis

	All Patients (*n* = 106)	
Characteristic	n	%
Age (years)		
≤ 60	59	55.7
> 60	47	44.3
Sex		
Male	42	39.6
Female	64	60.4
Location		
Colon	51	48.1
Rectum	55	51.9
Tumor grade		
Well or moderately differentiated	75	70.8
Poorly differentiated	31	29.2
Pathological T stage		
T1	5	4.7
T2	24	22.6
T3	52	49.1
T4	25	23.6
Pathological N stage		
N0	59	55.7
N1	20	18.9
N2	27	25.4
Pathological M stage		
M0	106	100

## Discussion

Despite increasing evidences that has indicated the critical roles of TRPV4 in the progression of various cancers, the potential involvement of TRPV4 in CRC metastasis is still not clear. Therefore, in the current study, we aimed to explore the underlying mechanism of TRPV4 controlling metastasis in colon cancer. Here, we showed that the inhibition of TRPV4 suppresseed invasiveness and overexpression of TRPV4-enhanced cell migration and invasion. Furthermore, we demonstrated that AKT-ZEB1 signaling-regulated EMT may be contributed to TRPV4-induced invasiveness (Fig. 4D). More importantly, we observed overexpression of TRPV4 and ZEB1 in patients with local metastasis, as well as a positive correlation between TRPV4 and ZEB1 expression.

Cumulative evidence has implicated that abnormal expression of the TRPV4 channel contributed to human cancer progression. Xie and colleagues suggested that TRPV4 served as preventive or therapeutic strategies for human gastric cancer [23]. Yu and colleagues reported that inhibition of TRPV4 reduced malignant biological behavior of hepatocellular carcinoma [24]. More recently, in a study carried out by our team, we demonstrated that TRPV4 was overexpressed in CRC and correlated with poor prognosis [13]. Moreover, endogenous TRPV4 expression was higher in HCT-116 and SW-620 cells and lower in HT-29 cells [13]. Consistent with the oncogenic properties of TRPV4, in this study we found that genetic knockdown or pharmacological inhibition of TRPV4 suppressed CRC cell migration and invasion. In addition, overexpression of TRPV4 promoted the migration and invasion capabilities of CRC cells. In line with our results, WH and colleagues suggested that TRPV4 dysfunction decreased aggression in breast cancer cells [12, 25]. Furthermore, Ou Yang-and colleagues proposed that TRPV4 accelerated glioma migration and invasion and served as a potential target for glioma therapy [26]. Therefore, we hypothesized that TRPV4 might be critical for CRC invasiveness, and that inhibition of TRPV4 could induce anti-metastasis in CRC.

Emerging evidence has suggested an important role of EMT in the metastatic process, which resulted in acquiring mesenchymal cell markers and losing epithelial markers [14–16]. Most solid cancers are of epithelial origin, and EMT has been considered an inducer for epithelial cells to acquire the capabilities of motility [14–16]. In a previous study, it was shown that E-cadherin regulated cell adhesion, and that low E-cadherin expression facilitated cells to detach from original sites [27]. On the contrary, N-cadherin and Vimentin are positively associated with increased cancer invasiveness [28, 29]. In the present study, inhibition of TRPV4 activity or expression enhanced the expression of E-cadherin and reduced the expression of N-cadherin and Vimentin. Notably, in support of the theory that TRPV4 positively regulated the EMT process, our results showed that overexpression of TRPV4 decreased the expression of E-cadherin and increased the expression of N-cadherin and Vimentin. It is well known that ZEB1 is a key activator of the EMT process in cancer cells [30]. Wang and colleagues suggested that ZEB1 promoted tumor metastasis through the EMT process in liver and breast cancer [22, 31]. In CRC, Su and colleagues proposed that ZEB1 might enhance EMT and metastasis. In line with this notion, we demonstrated that inhibition of TRPV4 decreased ZEB1 expression. Furthermore, ZEB1 knockdown may reverse the TRPV4-overexpression-induced EMT process and invasiveness. It was previously observed that AKT activation was involved in ZEB1 upregulation [32]. In this study, we found that AKT may play a key role in controlling TRPV4 regulated ZEB1 expression. More importantly, high expression of TRPV4 and ZEB1 was observed in CRC patients with local metastasis. We also indicated that in CRC patients, TRPV4 expression positively correlated with ZEB1 expression. Based on the important role of ZEB1 in EMT, we conclude that there might be a complex network involving TRPV4, ZEB1, EMT and invasiveness in CRC. Therefore, our findings strongly supported that the TRPV4/ZEB1 axis, as a key signaling that modulates EMT, may receive special attentive in future studies.

## Conclusions

Taken together, the results obtained in the present study showed that inhibition of TRPV4 suppressed CRC cell migration and invasion. We further uncovered that the invasiveness-inhibitory effect of TRPV4 inhibition was regulated by AKT-ZEB1 signaling through suppression of the EMT process. In this study, we highlighted the role of the TRPV4/ZEB1 axis in indicating EMT, which might hold potential for future diagnostic and therapeutic exploitation.

## Supplementary Information

**Additional file 1.**

## Data Availability

All data generated or analyzed during this study are included in this published article.
