# Supplementary material for: Identification of TRPV4 as a novel target in invasiveness of colorectal cancer
Source: BMC Cancer. 2021 Nov 23;21:1264. doi: 10.1186/s12885-021-08970-7 (PMC8611894; doi:10.1186/s12885-021-08970-7)
Supplement: Supplementary file 1 — Additional file 1. [file 12885_2021_8970_MOESM1_ESM.docx]

**Supplementary information**


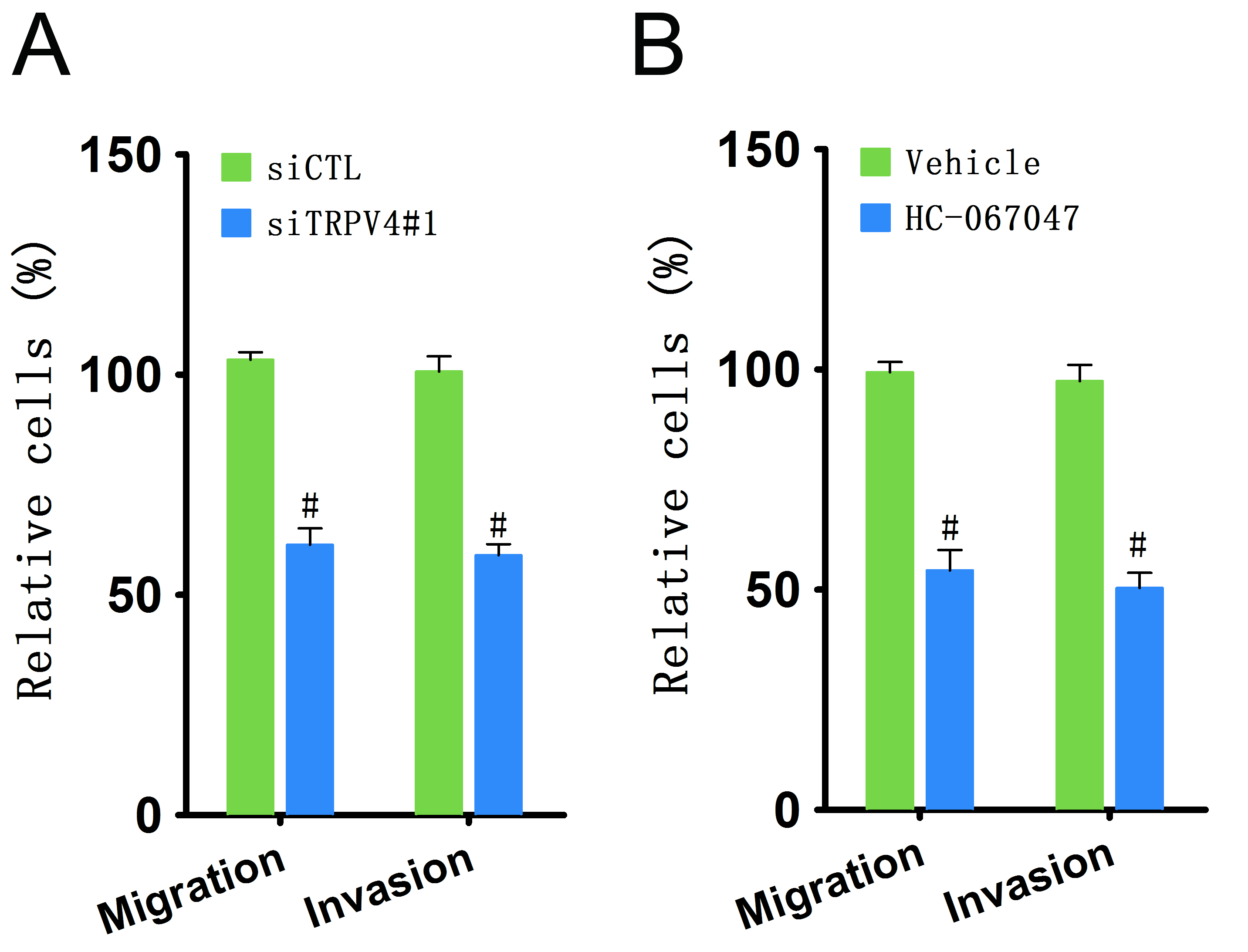


Figure S1. A, summary data of the migration and invasion assay in HT-29 cells transfected with siCTL and siTRPV4#1. B, summary data of the migration and invasion assay in HT-29 cells transfected with Vehicle or HC-067047. Values represent the mean ± SEM, #, p<0.05 compared to siCTL or Vehicle.


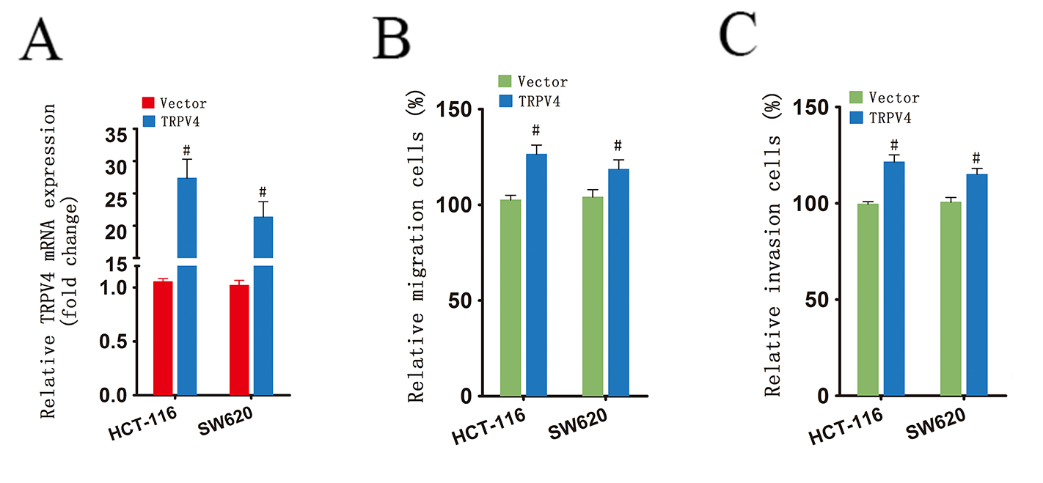


Figure S2. A, , TRPV4 mRNA levels in HCT-116 and SW620 cells transfected with a TRPV4-overexpression construct. B and C, Summary data from migration and invasion assay in HCT-116 cells and SW620 cells transfected with a TRPV4-overexpression construct. Values are means ± SEM, #, p<0.05 compared to Vector.


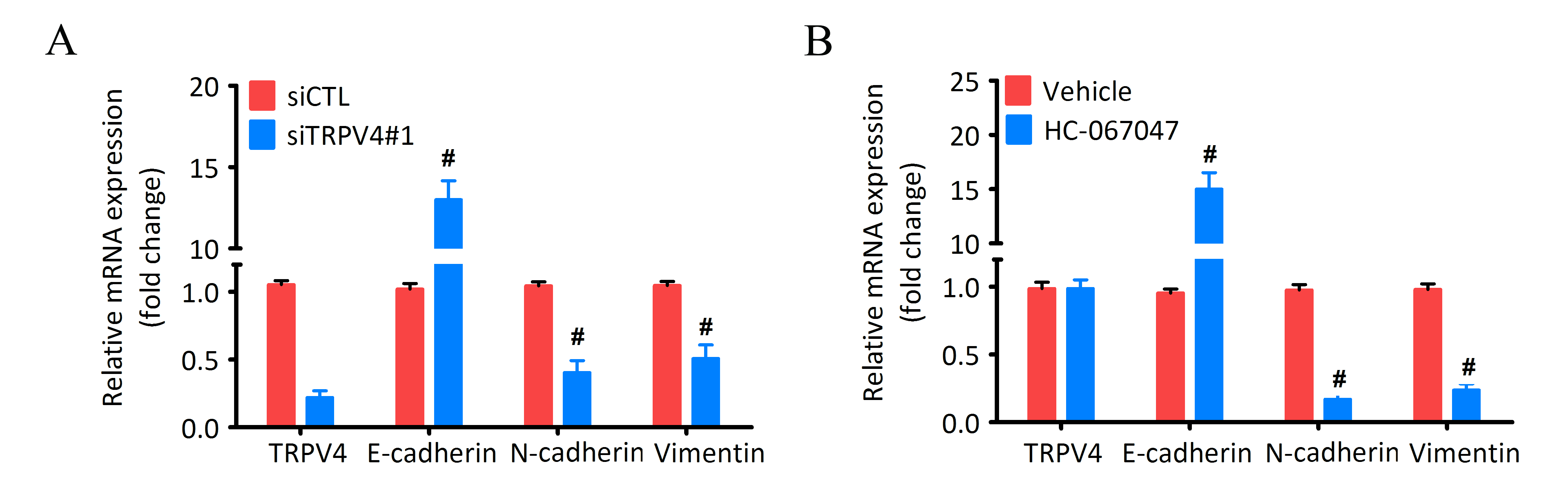


Figure S3. A and B, RNA levels of the epithelial-mesenchymal transition (EMT) markers in HT-29 cells transfected with siCTL or siTRPV4#1 or treated with Vehicle or HC-067047. Values are means ± SEM, #, p<0.05 compared to siCTL or Vehicle.


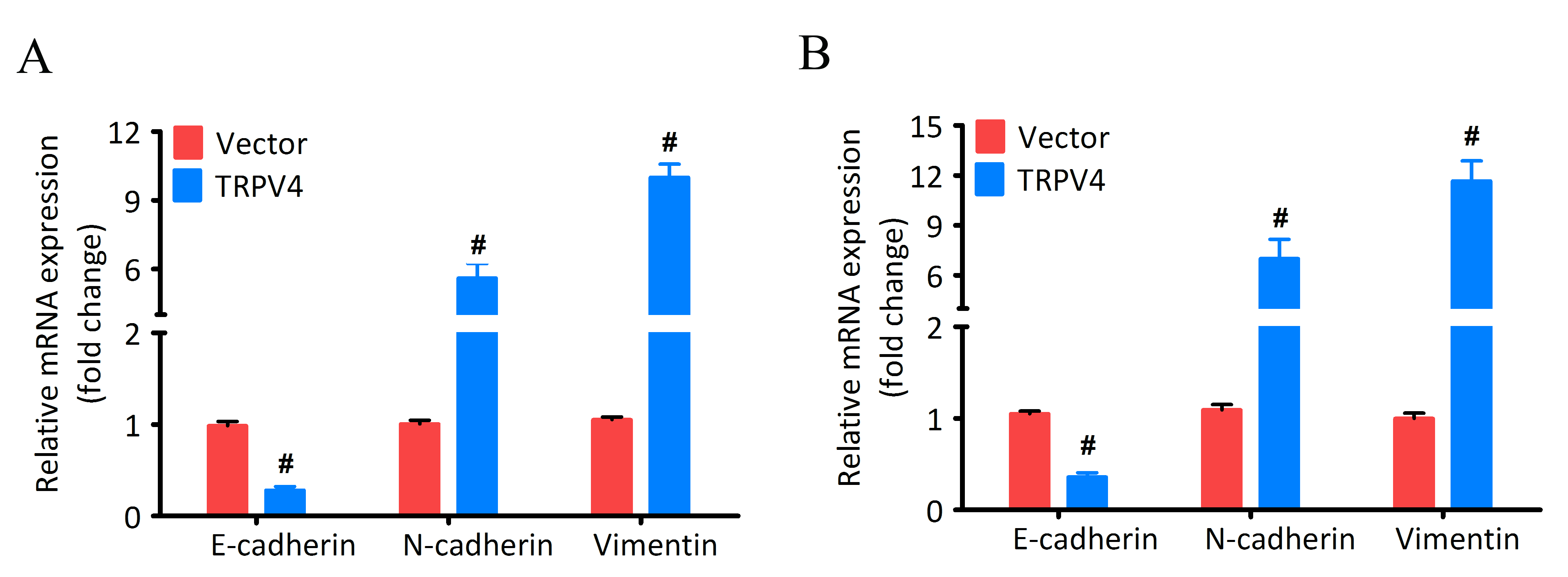


Figure S4. A, RNA levels of the epithelial-mesenchymal transition (EMT) markers in HCT-116 cells transfected with a TRPV4-overexpression construct. B, RNA levels of the EMT markers in SW620 cells transfected with a TRPV4-overexpression construct. Values are means ± SEM, #, p<0.05 compared to Vector.

Table S1 Primer sequences used for qPCR

| Gene Name | Primer | Sequence |
| --- | --- | --- |
| *TRPV4* | Forward  Reverse | TCACTCTCACCGCCTACTACCA CCCAGTGAAGAGCGTAATGAC |
| *E-cadherin* | Forward  Reverse | GCCTCCTGAAAAGAGAGTGGAAG  TGGCAGTGTCTCTCCAAATCCG |
| *N-cadherin* | Forward  Reverse | CCTCCAGAGTTTACTGCCATGAC  GTAGGATCTCCGCCACTGATTC |
| *Vimentin* | Forward  Reverse | AGGCAAAGCAGGAGTCCACTGA  ATCTGGCGTTCCAGGGACTCAT |
| *Actin* | Forward  Reverse | CACCATTGGCAATGAGCGGTTC  AGGTCTTTGCGGATGTCCACGT |
